# Predictors of childhood vaccination and recent influenza vaccination in older Brazilian adults: an analysis using conventional regression and machine learning approaches

**DOI:** 10.3389/fpubh.2026.1900872

**Published:** 2026-08-03

**Authors:** Xianglong Xu, Biying Wang, Kubra Maqsood Memon, Chen Sun, Yang Cheng, Fuquan Long

**Affiliations:** 1 Department of Sexually Transmitted Disease, Center of Infectious Skin Diseases, Shanghai Skin Disease Hospital, School of Medicine, Tongji University, Shanghai, China; 2 School of Public Health, Shanghai University of Traditional Chinese Medicine, Shanghai, China; 3 Artificial Intelligence and Modelling in Epidemiology Program, Melbourne Sexual Health Centre, Alfred Health, Carlton, VIC, Australia

**Keywords:** childhood, influenza, machine learning, older adults, predictors, recent vaccination, vaccination

## Abstract

**Background:**

Vaccination is an important public health intervention across the life course but its determinants may differ by life stage. We aimed to identify and compare predictors of childhood vaccination and recent influenza vaccination among older adults using conventional and machine learning approaches.

**Methods:**

We used data from the second wave (2019–2021) of the Brazilian Longitudinal Study of Aging (ELSI-Brazil), a nationally representative cohort of individuals aged 50+. Analyses included 9,211 participants for childhood vaccination and 9,863 for influenza vaccination. Predictors were selected from sociodemographic, environmental, lifestyle, and health domains. Multivariable logistic regression and machine learning algorithms were used, with performance evaluated via area under the receiver operating characteristic curve (AUC).

**Results:**

The best-performing machine learning model demonstrated acceptable performance for childhood vaccination (AUC = 0.72, 95% CI: 0.69–0.74) and recent influenza vaccination (AUC = 0.67, 95% CI: 0.64–0.70). For childhood vaccination, both analytical approaches identified age, sex, rural residence, parental education, school attendance at age 10, childhood access to books, and allergy as important predictors. Machine learning additionally identified age at school initiation, childhood economic status, self-rated childhood health, severe diarrhea, and history of childhood infectious diseases. Key predictors for recent influenza vaccination included age, marriage, educational level, employment, and hypertension/diabetes. Machine learning additionally highlighted the importance of vaccination history during childhood, life satisfaction, BMI, and mental health.

**Conclusion:**

Early-life socioeconomic and health conditions are important predictors of childhood immunization history, whereas current sociodemographic and health status are key predictors of recent vaccination. Machine learning identified supplementary predictors beyond traditional methods.

## 1 Introduction

Seasonal influenza remains a major global public health concern, contributing substantially to morbidity, mortality, and healthcare burden across populations (1). According to the World Health Organization fact sheet updated in 2025, seasonal influenza causes approximately 1 billion cases annually worldwide, including 3–5 million cases of severe illness and 290,000–650,000 respiratory deaths (2). Increasing flu vaccination coverage is therefore a key public health priority, as vaccination reduces the risk of severe disease, hospitalizations, and associated healthcare costs (3). However, flu vaccination uptake remains suboptimal in many populations and is influenced by a complex interplay of demographic, socioeconomic, behavioural, and health-related factors (4). Therefore, a deeper understanding of the factors that predict vaccination is essential for developing targeted, effective public health strategies to enhance vaccine uptake.

Older adults are a particularly critical target group for influenza vaccination due to their heightened vulnerability to severe complications, hospitalization, and death from the infection (5, 6). Despite facing elevated risks and receiving strong public health recommendations, vaccination coverage among older adults often remains insufficient. Understanding the specific determinants of vaccination in this age group is therefore a pressing public health priority. The challenge of protecting older adults is particularly salient in countries undergoing rapid demographic transitions, such as Brazil. As one of the world’s largest and most populous nations, Brazil is experiencing an accelerated aging process, with the proportion of its population aged 60 and over projected to increase dramatically in the coming decades (7). Thus, examining vaccination patterns within this unique and heterogeneous context is crucial for informing equitable public health policies tailored to older Brazilians.

Accurately identifying predictors for vaccination remains challenging using traditional analytic approaches, highlighting the need for advanced data-driven methods capable of capturing complex relationships within real-world population health data (8). Importantly, vaccination is not confined to a single life stage: early-life vaccination experiences (e.g., vaccination from birth to age 15) reflect childhood socioeconomic, environmental, and health conditions, whereas adult influenza vaccination represents current preventive decision-making (9). In our study, environmental variables were conceptualised as social and structural environments rather than physical or climatic exposures; for example, childhood household economic conditions and access to books may reflect early-life material resources and cognitive stimulation that shape long-term health literacy and preventive health behaviours (10). Consequently, a comprehensive understanding of vaccination behaviour requires examining both childhood and recent vaccination within a life-course framework (11).

Prediction models for influenza vaccination have proliferated in recent years, yet most focus exclusively on adult uptake using clinical or administrative data (12, 13). Using both traditional statistical approaches and machine learning methods, clinical prediction tools have demonstrated that routinely collected demographic and health-related variables can be integrated into risk-based frameworks to identify likely non-vaccinators (14). In large retrospective cohorts based on electronic medical records, multiple machine learning models have been applied to predict future influenza vaccination, with performance commonly evaluated using discrimination metrics such as the area under the receiver operating characteristic curve, illustrating the ability of machine learning methods to capture complex behavioural and healthcare-use patterns (15). Similarly, comparative studies have evaluated supervised learning approaches including logistic regression, support vector machines, random forests, and gradient boosting models for predicting influenza vaccination adherence, reflecting the growing interest in ensemble and non-linear methods for vaccination uptake prediction (8). Beyond individual-level clinical data, other work has explored alternative population and digital data sources, such as self-reported surveys and social media platforms, to predict influenza vaccination uptake, suggesting opportunities for broader surveillance while also raising concerns regarding representativeness and measurement validity (16). Despite these methodological advances, current models have limitations that restrict their usefulness in public health.

Previous models have been developed within specific healthcare systems or narrow subpopulations, which may limit their generalizability across diverse demographic and socioeconomic contexts (15). Some studies emphasize predictive accuracy while offering limited model interpretability, thereby constraining the translation of predictions into actionable public health interventions. In addition, heterogeneity in outcome definitions, predictor selection, and validation strategies complicates comparison across studies and reduces confidence in real-world scalability (8). These limitations highlight the need for interpretable, scalable prediction models using population-based data to inform vaccination strategies.

To address these gaps, our study aimed to identify and compare predictors of childhood vaccination and recent influenza vaccination among older adults through conventional and machine learning methodologies. Using a large, nationally representative dataset, we sought to develop models that are both predictive and interpretable, thereby providing insights for public health practice.

## 2 Methods

### 2.1 Study design and data source

We used data from the second wave (2019–2021) of the Brazilian Longitudinal Study of Aging (ELSI-Brazil), a nationally representative cohort of individuals aged ≥50 years, covering residents from 70 municipalities across five regions of Brazil. Our study aimed to analyse vaccination history from birth to age 15 and influenza vaccination in the past 12 months, along with their associated factors. Inclusion criteria were age ≥50 years; exclusion criteria were: (1) missing information on vaccination from birth to age 15, or (2) missing information on influenza vaccination in the past 12 months. A total of 9,211 participants were included for the analysis of vaccination from birth to age 15 and its determinants, and 9,863 participants for the analysis of influenza vaccination in the past 12 months and its determinants. ELSI-Brazil was approved by the Research Ethics Committee of the Oswaldo Cruz Foundation, René Rachou Institute (protocol number CAAE 34649814.3.0000.5091). All participants provided written informed consent.

Our study adhered to the Strengthening the Reporting of Observational Studies in Epidemiology (STROBE) checklist for cross-sectional studies (17) and the Transparent Reporting of a multivariable prediction model for Individual Prognosis Or Diagnosis (TRIPOD) statement for prediction model development and validation (18).

### 2.2 Predictors and outcome

In our study, the outcome variables were dichotomous: (1) childhood vaccination, indicating receipt of any vaccine from birth to age 15 (Yes/No), and (2) recent influenza vaccination, indicating receipt of an influenza vaccine in the past 12 months (Yes/No). The childhood vaccination variable aimed to assess early-life immunization history, while recent influenza vaccination reflected current preventive behaviour; these variables were analysed separately. Predictors were classified into sociodemographic, environmental, lifestyle, and disease-related factors, determined in advance based on item availability in the ELSI-Brazil questionnaire. Environmental factors referred to individual-level social and structural conditions, including self-reported childhood household economic conditions, access to books, and perceived discrimination when seeking medical services. All environmental variables used in this study (e.g., having books at home, economic situation until age 15) are individual-level characteristics based on participants’ self-reported childhood experiences, not community-level aggregate measures. A comprehensive list of predictors can be found in Table 1.

**Table 1:** Predictors for vaccination among adults aged 50 years or above in ELSI-Brazil.

Variable type	Variables	
	Outcome 1: Childhood vaccination	Outcome 2: Recent vaccination
Sociodemographic characteristics	Age; Sex; Rural residence until 15; Mother’s educational level; Father’s educational level; Age when starting school; Schooling at age 10;	Age; Sex; Rural residence until age 15; Marriage; Number of children; Mother’s educational level; Father’s educational level; Educational background; Economic situation (relative to before adulthood); Have a monetary job (within 30 days); Life satisfaction(income/work); Health condition; Have friends
Environmental factors	Living with family (at age 10); Had books at home (at age 10); Economic situation (until age 15)	Discrimination (when seeking medical services); Economic situation (until age 15)
Lifestyle factors	Health condition (until age 15); Absent from school due to health issues (until age 15)	Exercise frequency; Alcohol intake frequency; Smoke frequency; Frequency of poor mental health; Sleep quality; Sleep problems (last 6 months); Optimistic about the future
Disease factors	Respiratory diseases (until age 15); Allergy (until age 15); Chronic ear problems (until age 15); Common childhood infectious diseases (until age 15); Poliomyelitis (until age 15); Severe diarrhea (typhoid or other until age 15)	Respiratory diseases (until age 15); Allergy (until age 15); Common childhood infectious diseases (until age 15); Poliomyelitis (until age 15); Typhoid (until age 15); Vaccination (until age 15); Hypertension; Diabetes; Depression; Cancer
Physical examinations	—	BMI

### 2.3 Data analysis

#### 2.3.1 Statistical analyses

Descriptive analyses presented categorical variables as frequencies and percentages, while continuous variables were summarized as median (interquartile range). For each vaccination outcome, univariable and multivariable logistic regression models were fitted. Univariable models estimated crude odds ratios (ORs) with 95% confidence intervals (CIs) for each predictor individually. Multivariable models included all prespecified covariates simultaneously to obtain adjusted ORs and corresponding CIs. Reference levels for categorical variables followed the dataset’s predefined baseline and were implemented using dummy coding; continuous and ordinal variables were interpreted per-unit change. To address potential complete separation or convergence failure, penalized logistic regression with L2 regularization was automatically applied when standard maximum-likelihood estimation failed to converge. Statistical significance was set at two-sided *p* < 0.05. All regression results were visualized using forest plots to display effect directions and uncertainty.

#### 2.3.2 Machine learning model development

Data processing and modelling were performed in R 4.2.1. Data preprocessing strictly followed the ELSI-Brazil codebook and variable definitions: sparse categories were merged according to prespecified rules, numerical features were normalized, and categorical features were numerically encoded. The dataset was split into 80% training and 20% testing sets via stratified random sampling, with the former used for model development and the latter for independent validation. Variables with >30% missing values were excluded, while variables with ≤30% missingness were imputed using predictive mean matching (PMM) via multiple imputation with the mice package. To address class imbalance in machine-learning model development, the training set was balanced using random undersampling of the majority class (19) to reduce majority-class dominance during model training, while the testing set retained the original distribution to ensure realistic evaluation of generalization performance and clinical applicability.

Machine learning model development was performed using the H2O automated machine learning (AutoML) framework, enabling parallel training and selection of multiple algorithms. The algorithm library included generalized linear models (GLM), gradient boosting machines (GBM), distributed random forests (DRF), multilayer perceptron (MLP), and stacked ensemble models (StackedEnsemble) built from the base learners. The stacking strategy used cross-validated predictions from base learners as meta-features to train a second-level meta-learner (default GLM), aiming to combine heterogeneous algorithm strengths and reduce bias from any single model. During model training, H2O AutoML performed 3-fold cross-validation with the area under the receiver operating characteristic curve (AUC) as the primary metric for early stopping and model ranking (stopping_metric and sort_metric both set to AUC). The top-ranked model on the leaderboard was selected as the final model.

#### 2.3.3 Model performance evaluation and validation

A multidimensional evaluation framework was constructed focusing on discrimination and calibration. Discrimination was assessed using AUC, accuracy, sensitivity, specificity, and confusion-matrix-derived metrics. Given the class-imbalanced nature of the outcomes, the precision-recall curve (PR curve) and its area under the curve (AUPRC) were additionally reported to comprehensively evaluate performance on the minority class. When interpreting the AUC value, an AUC > 0.6 indicates acceptable discrimination (20).

#### 2.3.4 Model ranking rules

In the conventional statistical analysis, key predictors were identified from multivariable logistic regression based on adjusted odds ratios (aORs) and 95% confidence intervals (CIs), with a two-sided *p* < 0.05 considered statistically significant. In the machine learning analysis, feature importance was quantified using the built-in variable importance measure embedded in each eligible model from the H2O AutoML platform. For each algorithm, all predictors were ranked in descending order of model-specific relative importance, where rank 1 indicated the most predictive variable. Raw importance scores were not compared across models, because their underlying calculation methods and numerical scales differ between paradigms. Therefore, we used within-model relative rankings to allow consistent comparisons across different learners. We derived two aggregate metrics to synthesise results across all machine learning models: Best ML rank, defined as the smallest numerical rank achieved by a given predictor across all eligible models; and Top10 models, defined as the number of models in which the predictor ranked in the top 10 for importance. Finally, all predictors were categorised into four groups according to their concordance across the two analytical paradigms: Concordant (statistically significant in logistic regression and ranked in the top 10 in at least one machine learning model), Inference-only (statistically significant in logistic regression but not ranked in the top 10 in any machine learning model), Prediction-only (non-significant in logistic regression but ranked in the top 10 in at least one machine learning model), and Neither (non-significant in both paradigms).

## 3 Results

### 3.1 Characteristics of the study population

Table 2 displays the characteristics of the study population categorized by their vaccination status over two distinct time frames. Among those recently vaccinated, 71.4% (7,040/9863) had received an influenza vaccine within the previous year. The median age was 67 years, with 60.9% (4,288/7040) being male and 39.1% (2,752/7040) female. In the childhood vaccination group, 63.5% (5,848/9211) had received at least one vaccine between birth and age 15. This group consisted of 58.2% (3,403/5848) male and 41.8% (2,445/5848) female participants, with an median age of 62 years. Within the recent vaccination cohort, 74.2% (7,317/9863) were unemployed, and 53.2% (5,251/9863) were married. Additional characteristics were provided in Supplementary Table 1, while Table 1 in the main text provides an overview of selected key variables.

**Table 2:** Characteristics of study participants, stratified by vaccination.

Characteristics	Recent vaccination (*n* = 9,863)		Childhood vaccination (*n* = 9,211)	
	Unvaccinated (*n* = 2,823)	Vaccinated (*n* = 7,040)	Unvaccinated (*n* = 3,363)	Vaccinated (*n* = 5,848)
Age	59 (55–68)	67 (60–75)	69 (61–77)	62 (57–70)
Sex				
Male	1,557 (55.2%)	4,288 (60.9%)	2,063 (61.3%)	3,403 (58.2%)
Female	1,266 (44.8%)	2,752 (39.1%)	1,300 (38.7%)	2,445 (41.8%)
Marriage				
Single/divorced or separated/widow (er)	1,258 (44.6%)	3,354 (47.6%)	NA	NA
Married/common-law marriage/live together	1,565 (55.4%)	3,686 (52.4%)	NA	NA
Have a monetary job (within 30 days)				
No	1,762 (62.4%)	5,555 (78.9%)	NA	NA
Yes	1,061 (37.6%)	1,485 (21.1%)	NA	NA
Hypertension				
No	1,628 (57.7%)	3,124 (44.4%)	NA	NA
Yes	1,195 (42.3%)	3,916 (55.6%)	NA	NA
Diabetes				
No	2,478 (87.8%)	5,605 (79.6%)	NA	NA
Yes	345 (12.2%)	1,435 (20.4%)	NA	NA
Common childhood infectious diseases (until age 15)				
No	505 (17.9%)	1,137 (16.2%)	689 (20.5%)	851 (14.6%)
Yes	2,318 (82.1%)	5,903 (83.8%)	2,674 (79.5%)	4,997 (85.4%)
Vaccination (until age 15)				
No	894 (31.7%)	2,750 (39.1%)	NA	NA
Yes	1,929 (68.3%)	4,290 (60.9%)	NA	NA

Median (Q_1_, Q_3_); Common childhood infectious diseases include measles, rubella, chickenpox, mumps, diphtheria, whooping cough, and scarlet fever; NA, not applicable.

### 3.2 Class distribution before and after training-set balancing

For childhood vaccination, the overall analytic sample included 5,848 vaccinated participants (63.5%) and 3,363 unvaccinated participants (36.5%). After stratified splitting, the original training set included 4,679 vaccinated and 2,691 unvaccinated participants. After training-set class balancing, the distribution was 2,691 vaccinated and 2,691 unvaccinated participants. The testing set was not resampled and retained the original distribution, with 1,169 vaccinated and 672 unvaccinated participants.

For recent influenza vaccination, the overall analytic sample included 7,040 vaccinated participants (71.4%) and 2,823 unvaccinated participants (28.6%). The original training set included 5,632 vaccinated and 2,259 unvaccinated participants. After class balancing, the training set included 2,259 vaccinated and 2,259 unvaccinated participants. The testing set remained untouched, with 1,408 vaccinated and 564 unvaccinated participants.

### 3.3 Prediction performance of childhood vaccination and recent influenza vaccination

**Figure 1:**
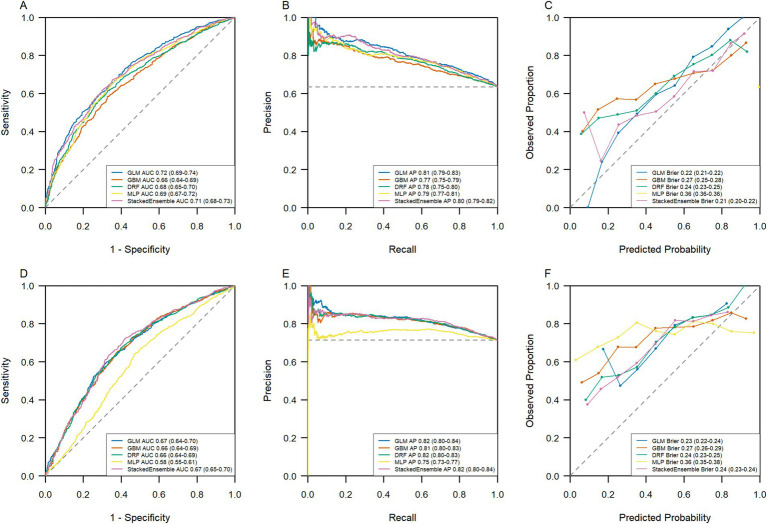
Predictive performance of machine learning models for childhood **(A–C)** and recent **(D–F)** vaccination outcomes. ROC curves **(A)**, precision-recall (PR) curves **(B)**, and calibration curves **(C)** for childhood vaccination models. ROC curves (D), precision-recall (PR) curves **(E)**, and calibration curves **(F)** for recent vaccination models. AUC, area under the curve; AP, area under the precision-recall curve; PR, precision-recall.

In the context of childhood vaccination prediction, five machine learning models (GLM, GBM, DRF, MLP, and Stacked Ensemble) were assessed (Figures 1A–C). The GLM model exhibited the highest performance with an AUC of 0.72 (95% CI: 0.69–0.74), followed by Stacked Ensemble (AUC = 0.71, 95% CI: 0.68–0.73), MLP (AUC = 0.69, 95% CI: 0.67–0.72), DRF (AUC = 0.68, 95% CI: 0.65–0.70), and GBM (AUC = 0.66, 95% CI: 0.64–0.69).

Regarding the prediction of recent influenza vaccination (Figures 1D–F), the GLM and Stacked Ensemble models demonstrated superior performance, with AUCs of 0.67 (95% CI: 0.64–0.70) and 0.67 (95% CI: 0.65–0.70), respectively, followed by GBM and DRF with an AUC of 0.66 (95% CI: 0.64–0.69, 95% CI: 0.64–0.69). MLP exhibited the lowest performance with an AUC of 0.58 (95% CI: 0.55–0.61).

### 3.4 Predictors of childhood vaccination and recent influenza vaccination

The machine-learning rankings shown in Tables 3, 4 were based on the predefined model-specific feature-importance procedure described in the Methods; therefore, predictors were compared according to their within-model ranks rather than raw importance scores.

**Table 3:** Predictors of childhood vaccination identified by conventional multivariable logistic regression and machine learning variable importance rankings.

Variable Type	Predictor	aOR (95% CI), *p* value	ML rank (GLM/ GBM/ DRF/MLP)	Best ML rank	Top10 models	Category
Sociodemographic	Age	0.96 (0.95–0.96), *p* < 0.001	1/1/1/1	1 (GLM)	4/4	Concordant
	Sex	1.14 (1.04–1.26), *p* = 0.005	17/8/8/8	8 (GBM)	3/4	Concordant
	Rural residence until 15	0.75 (0.67–0.83), *p* < 0.001	7/10/11/7	7 (GLM)	3/4	Concordant
	Mother’s educational level: Completed elementary school	1.81 (1.48–2.20), *p* < 0.001	6/6/6/4	4 (MLP)	4/4	Concordant
	Father’s educational level: Incomplete elementary school	1.22 (1.07–1.39), *p* = 0.003	8/5/5/14	5 (GBM)	3/4	Concordant
	Age when starting school	1.00 (0.99–1.00), *p* = 0.429	15/2/2/3	2 (GBM)	3/4	Prediction-only
	Schooling at age 10	1.69 (1.49–1.91), *p* < 0.001	2/9/7/6	2 (GLM)	4/4	Concordant
Environmental	Lived with family (at age 10)	1.07 (0.86–1.33), *p* = 0.552	16/16/16/16	16 (GLM)	0/4	Neither
	Had books at home (at age 10)	1.48 (1.33–1.64), *p* < 0.001	4/12/10/13	4 (GLM)	2/4	Concordant
	Economic situation (until age 15): Poor	0.81 (0.63–1.03), *p* = 0.09	18/4/4/9	4 (GBM)	3/4	Prediction-only
Lifestyle	Health condition (until age 15): Good	0.93 (0.82–1.04), *p* = 0.189	14/3/3/10	3 (GBM)	3/4	Prediction-only
	Absent from school due to health issues (until age 15): Did not go to school until 15	0.85 (0.74–0.98), *p* = 0.021	10/7/9/5	5 (MLP)	4/4	Concordant
Disease	Common childhood infectious diseases (until age 15)	1.06 (0.88–1.27), *p* = 0.541	3/11/12/18	3 (GLM)	1/4	Prediction-only
	Poliomyelitis (until age 15)	1.37 (1.15–1.64), *p* < 0.001	11/18/18/2	2 (MLP)	1/4	Concordant
	Respiratory diseases (until age 15)	1.15 (0.92–1.43), *p* = 0.219	12/15/15/15	12 (GLM)	0/4	Neither
	Allergy (until age 15)	1.64 (1.46–1.86), *p* < 0.001	9/14/14/17	9 (GLM)	1/4	Concordant
	Severe diarrhea (typhoid or other until age 15)	--	5/13/13/12	5 (GLM)	1/4	Prediction-only
	Chronic ear problems (until age 15)	1.55 (1.33–1.81), *p* < 0.001	13/17/17/11	11 (MLP)	0/4	Inference-only

Predictors are categorized based on agreement between logistic regression (LR) and machine learning (ML) models:aOR:adjusted odds ratios; CI, confidence interval; GLM, generalized linear model; GBM, gradient boosting machine; DRF, distributed random forest; MLP, multilayer perceptron. ML ranks were determined using the built-in feature-importance output from each eligible H2O machine-learning model. The “ML rank” column presents the within-model rank of each predictor in the order of the models listed in the table. “Best ML rank” refers to the smallest numerical rank achieved by a predictor across all eligible machine-learning models, with the corresponding model shown in parentheses. “Top10 models” indicates the number of eligible models in which the predictor appeared among the top 10 important variables. Concordant: Statistically significant in LR (*p* < 0.05) AND ranked in top 10 by at least one ML model. Inference-only: Statistically significant in LR but NOT ranked in top 10 by any ML model. Prediction-only: Not significant in LR (or excluded) but ranked in top 10 by at least one ML model. Neither: Not significant in LR and not ranked in top 10 by any ML model.

**Table 4:** Predictors of recent vaccination identified by conventional multivariable logistic regression and machine learning variable importance rankings.

Variable type	Predictor	aOR (95% CI), *p* value	ML rank (GLM/GBM/DRF)	Best ML rank	Top10 models	Category
Sociodemographic	Age	1.06 (1.05–1.06), *p* < 0.001	1/1/1	1 (GLM)	3/3	Concordant
	Sex	0.84 (0.76–0.93), *p* < 0.001	28/19/20	19 (GBM)	0/3	Inference-only
	Rural residence until age 15	--	29/18/19	18 (GBM)	0/3	Neither
	Marriage: married/common-law marriage/live together	1.29 (1.12–1.50), *p* < 0.001	13/10/9	9 (DRF)	2/3	Concordant
	Number of children	0.97 (0.95–0.99), *p* = 0.009	17/4/4	4 (GBM)	2/3	Concordant
	Mother’s educational level: completed middle school	1.57 (1.17–2.11), *p* = 0.003	5/13/10	5 (GLM)	2/3	Concordant
	Father’s educational level: completed elementary school	--	31/11/13	11 (GBM)	0/3	Neither
	Educational background: completed high school	1.31 (1.14–1.51), *p* < 0.001	16/7/8	7 (GBM)	2/3	Concordant
	Economic situation (relative to before adulthood): got worse	--	32/17/18	17 (GBM)	0/3	Neither
	Have a monetary job(within 30 days)	0.67 (0.60–0.75), *p* < 0.001	2/15/15	2 (GLM)	1/3	Concordant
	Life satisfaction (income/work)	1.02 (1.00–1.04), *p* = 0.067	27/3/3	3 (GBM)	2/3	Prediction-only
	Health condition: very bad	--	12/8/7	7 (DRF)	2/3	Prediction-only
	Have friends	--	20/28/27	20 (GLM)	0/3	Neither
Environmental	Discrimination (when seeking medical services)	--	26/30/30	26 (GLM)	0/3	Neither
	Economic situation (until age 15): was varied a lot	--	23/12/11	11 (DRF)	0/3	Neither
Lifestyle	Exercise frequency	1.04 (0.91–1.18), *p* = 0.583	11/6/6	6 (GBM)	2/3	Prediction-only
	Alcohol intake frequency: once a month or more	0.79 (0.69–0.91), *p* < 0.001	6/16/16	6 (GLM)	1/3	Concordant
	Frequency of poor mental health	0.99 (0.99–1.00), *p* = 0.038	4/9/12	4 (GLM)	2/3	Concordant
	Sleep quality: regular	1.23 (1.06–1.42), *p* = 0.005	15/5/5	5 (GBM)	2/3	Concordant
	Sleep problems (last 6 months)	0.87 (0.76–0.99), *p* = 0.038	30/24/22	22 (DRF)	0/3	Inference-only
	Optimistic about the future: always	1.37 (1.12–1.67), *p* = 0.002	10/14/14	10 (GLM)	1/3	Concordant
Disease	Respiratory diseases (until age 15)	1.22 (1.02–1.46), *p* = 0.029	22/29/29	22 (GLM)	0/3	Inference-only
	Allergy (until age 15)	--	18/25/28	18 (GLM)	0/3	Neither
	Common childhood infectious diseases (until age 15)	1.03 (0.91–1.16), *p* = 0.666	21/22/23	21 (GLM)	0/3	Neither
	Poliomyelitis(until age 15)	0.72 (0.48–1.07), *p* = 0.102	14/32/32	14 (GLM)	0/3	Neither
	Severe diarrhea(until age 15)	--	24/26/26	24 (GLM)	0/3	Neither
	Vaccination(until age 15)	0.91 (0.82–1.00), *p* = 0.06	9/21/21	9 (GLM)	1/3	Prediction-only
	Hypertension	1.35 (1.23–1.49), *p* < 0.001	3/20/17	3 (GLM)	1/3	Concordant
	Diabetes	1.56 (1.37–1.79), *p* < 0.001	8/23/24	8 (GLM)	1/3	Concordant
	Depression	--	7/27/25	7 (GLM)	1/3	Prediction-only
	Cancer	1.09 (0.86–1.39), *p* = 0.471	19/31/31	19 (GLM)	0/3	Neither
Physical exams	BMI	--	25/2/2	2 (GBM)	2/3	Prediction-only

Predictors are categorized based on agreement between logistic regression (LR) and machine learning (ML) models; aOR, adjusted odds ratios; CI, confidence interval; GLM, generalized linear model; GBM, gradient boosting machine; DRF, distributed random forest. ML ranks were determined using the built-in feature-importance output from each eligible H2O machine-learning model. The “ML rank” column presents the within-model rank of each predictor in the order of the models listed in the table. “Best ML rank” refers to the smallest numerical rank achieved by a predictor across all eligible machine-learning models, with the corresponding model shown in parentheses. “Top10 models” indicates the number of eligible models in which the predictor appeared among the top 10 important variables. Concordant: Statistically significant in LR (*p* < 0.05) AND ranked in top 10 by at least one ML model. Inference-only: Statistically significant in LR but NOT ranked in top 10 by any ML model. Prediction-only, not significant in LR (or excluded) but ranked in top 10 by at least one ML model. Neither, not significant in LR and not ranked in top 10 by any ML model.

Table 3 showed the predictors of childhood vaccination identified by multivariable logistic regression and machine learning algorithms. Key predictors consistently identified by both methods include: age, sex, rural residence, parental education, school attendance, access to books during childhood, allergy, and absence from school due to health issues. These variables were statistically significant in logistic regression and were also ranked among the top 10 important predictors in at least one ML model. Notably, ML algorithms identified several predictors not found significant in logistic regression, including age at starting school, economic status, health condition, history of common childhood infectious diseases, and severe diarrhea. Conversely, logistic regression singled out chronic ear problems as a significant predictor, although it was not prioritized by any ML model. Factors such as childhood respiratory diseases and living with family at age 10 did not reach significance in either approach.

Table 4 presented the predictors of recent vaccination in older adults, as identified by both multivariable logistic regression and machine learning algorithms. Both analytical approaches consistently identified several key predictors as significant including age, marriage, educational level, number of children, having a paid job, alcohol intake, mental health, sleep quality, optimistic about the future, and having hypertension or diabetes. These factors were statistically significant in the regression model and were also ranked among the top 10 most important predictors by at least one machine learning model. Machine learning also identified important predictors such as life satisfaction, BMI, and vaccination history during childhood, which were not statistically significant in the logistic regression model. Conversely, logistic regression identified factors like sex and sleep problems as significant predictors, though they were not prioritized by the machine learning models.

## 4 Discussion

Our study identified key predictors of vaccination among adults aged 50 years and older using conventional and machine learning approaches. We focused on two distinct life-course measures: childhood vaccination, reflecting early-life socioeconomic and health conditions, and recent influenza vaccination, capturing preventive health behaviour in later adulthood (10, 11). Our models demonstrated acceptable predictive performance for both outcomes, achieving the study’s objective. Through a nationally representative cross-sectional survey, we analysed a large dataset and identified important factors influencing vaccination in this age group, providing evidence that may inform public health policy and targeted intervention strategies. Collectively, these findings highlight how vaccination in later life is influenced by both early-life and contemporary factors, reinforcing the importance of a life-course approach to understanding and promoting vaccine uptake.

Our findings reaffirm key socioeconomic and demographic determinants of vaccination established in prior literature. Furthermore, by applying machine learning, we uncovered previously under-recognised early-life predictors. These factors exhibit complex, nonlinear, or interactive relationships with immunization uptake—relationships that conventional methods may overlook. Maternal primary education level emerged as the strongest positive predictor of childhood vaccination, underscoring the decisive role of caregivers’ health literacy and resource access—a finding consistent with existing evidence across diverse contexts (21, 22). This reinforces the need for interventions tailored to families with lower maternal education, such as community-based health education, free vaccine distribution, or integrated parent–child vaccination incentives.

Machine learning identified several early-life predictors of childhood vaccination that operate through indirect or interactive pathways rather than direct effects. For early vaccination, these included school-entry age, childhood economic status, self-rated childhood health, and history of polio—variables likely operating through indirect or interacting pathways such as delayed social integration, cumulative socioeconomic disadvantage, or health-related vulnerability. School-entry age, for instance, may serve as a proxy for developmental delays or later social integration, with effects potentially amplified through complex interactions with other determinants. Similarly, the predictive value of school attendance at age 10 and household book resources supports the importance of early social integration and an enriched cognitive environment, pointing to schools as key settings for vaccination programmes (23, 24). These findings suggest that early-life social and structural environments may influence vaccination uptake by shaping access to health information, caregiver health literacy, school engagement, and preventive health norms across the life course. Furthermore, gender differences in vaccination emerged across the life course. Moreover, a history of childhood infectious diseases or severe diarrhea was associated with early vaccination.

Our study confirms that adult influenza vaccination is shaped by multifactorial determinants beyond conventional sociodemographic and clinical risks. Using machine learning, we identified significant predictive roles for interconnected physiological, psychological, and social well-being factors. While being employed was associated with lower vaccine uptake—highlighting persisting time and access barriers that could be mitigated through workplace vaccination services or flexible scheduling (25, 26) —higher uptake among individuals with hypertension or diabetes aligns with established evidence linking chronic disease management to greater health service engagement and provider recommendation (27, 28). This pattern, however, points to a concerning “prevention gap” among healthier adults who may perceive less need for vaccination, indicating that public health messaging must counteract the misconception that influenza vaccination is only necessary for those with existing medical conditions.

Beyond traditional determinants, our analysis identifies modifiable psychosocial factors that offer new targets for personalised vaccination promotion. Notably, machine learning approaches identified several predictors not emphasised in conventional methods, including life satisfaction, current self-rated health, exercise frequency, BMI, and frequency of psychological distress. These variables suggest that recent influenza vaccination is shaped by multidimensional and often non-linear interactions between physical fitness, mental well-being, and subjective health perception—relationships that linear models may fail to fully capture. For instance, the positive association with an optimistic attitude supports incorporating psychological factors into health promotion, potentially via community-based positive psychology interventions (29). Together, these insights offer new levers for more precise vaccination promotion, moving beyond one-size-fits-all campaigns to strategies that account for an individual’s holistic health profile and life context.

Our findings have some implications for the design of vaccination programmes across the life course. For childhood immunisation, the substantial influence of early-life socioeconomic and educational determinants underscores the necessity of reinforcing family- and school-centred interventions. Evidence-based models may include strengthening school-based delivery systems, improving targeted health literacy among caregivers, and prioritising outreach to children from disadvantaged backgrounds (30). In contrast, influenza vaccination in adulthood appears more immediately constrained by structural and health-related barriers, indicating that strategies to enhance convenience and access—such as workplace vaccination services, extended clinic hours, or streamlined appointment systems—could yield significant uptake improvements (28). Moreover, the identification of multidimensional predictors through machine learning highlights the potential of data-enabled precision in vaccination campaigns. Risk-stratification approaches could support more tailored outreach and efficient resource allocation, moving beyond blanket strategies toward differentiated promotion. Collectively, these insights advocate for life-course-informed vaccination policies that address both foundational early-life determinants and contemporary adult barriers—an approach particularly pertinent for supporting ageing populations in low- and middle-income settings, where systemic constraints and epidemiological transitions converge.

Our study has several limitations that should be considered when interpreting the findings. First, the cross-sectional nature of the data precludes causal inference between the identified predictors and vaccination outcomes. Second, all measures were based on self-report, which may introduce recall bias and social-desirability effects. Third, the sample was limited to adults aged 50 years and older in Brazil; thus, the results may not be generalizable to younger populations or to other national contexts. Fourth, our models did not include direct measures of public health policies, local immunization campaigns, regional healthcare infrastructure, or temporal changes in Brazilian influenza vaccination recommendations because these contextual factors were not available in the dataset, although they may have influenced vaccination uptake during the study period. Future research should integrate such contextual variables, potentially through linkage with administrative or regional health system data, to improve predictive performance and policy relevance. Finally, although internal validation suggested acceptable predictive performance, external validation in independent Brazilian cohorts is needed before these models can be applied in clinical or public health practice.

## 5 Conclusion

By integrating conventional and machine learning approaches within a life-course framework, our study identified distinct determinants of vaccination across the lifespan of older adults. Childhood vaccination was primarily shaped by early-life socioeconomic and developmental factors, whereas recent influenza vaccination was driven more by contemporary sociodemographic, health, and psychosocial conditions. Using a large, nationally representative cohort, our analysis offers a population-level perspective on vaccine uptake in an ageing society. These findings provide an understanding of vaccination as a lifelong health behaviour and highlight the potential for tailored, stage-specific interventions to improve vaccine coverage across the life course.

## Data Availability

The datasets presented in this study can be found in online repositories. The names of the repository/repositories and accession number(s) can be found at: https://elsi.cpqrr.fiocruz.br/ ELSI-Brazil (Estudo Longitudinal da Saúde dos Idosos Brasileiros).
