# Supplementary material for: Predictors of childhood vaccination and recent influenza vaccination in older Brazilian adults: an analysis using conventional regression and machine learning approaches
Source: Front Public Health. 2026 Aug 3;14:1900872. doi: 10.3389/fpubh.2026.1900872 (PMC13477924; doi:10.3389/fpubh.2026.1900872)
Supplement: Supplementary file 1 [file Table_1.docx]

**Supplementary Table 1**

Characteristics of study participants stratified by childhood vaccination.

| **Characteristic** | **childhood vaccination** | |
| --- | --- | --- |
|  | **Unvaccinated**  **(n = 3,363)** | **Vaccinated**  **(n = 5,848)** |
| **Age** | 69 (61-77) | 62 (57-70) |
| **Sex** | 2,063(61.3%) | 3,403(58.2%) |
| Male | 2,063(61.3%) | 3,403(58.2%) |
| Female | 1,300(38.7%) | 2,445(41.8%) |
| **Rural residence until 15** |  |  |
| No | 1,102(32.8%) | 2,872(49.1%) |
| Yes | 2,261(67.2%) | 2,976(50.9%) |
| **Mother's educational level** |  |  |
| Illiterate/never studied | 2,402(71.4%) | 3,041(52.0%) |
| Incomplete elementary school | 601(17.9%) | 1,463(25.0%) |
| Completed elementary school | 210(6.2%) | 889(15.2%) |
| Completed middle school | 87(2.6%) | 204(3.5%) |
| High school or above | 63(1.9%) | 251(4.3%) |
| **Father's educational level** |  |  |
| Illiterate/never studied | 2,182(64.9%) | 2,756(47.1%) |
| Incomplete elementary school | 667(19.8%) | 1,563(26.7%) |
| Completed elementary school | 288(8.6%) | 903(15.4%) |
| Completed middle school | 92(2.7%) | 239(4.1%) |
| High school or above | 134(4.0%) | 387(6.6%) |
| **Age when starting school** | 7 (4-7) | 7 (7-7) |
| **Living with family (at age 10)** |  |  |
| No | 167(5.0%) | 242(4.1%) |
| Yes | 3,196(95.0%) | 5,606(95.9%) |
| **Schooling at age 10** |  |  |
| No | 1,462(43.5%) | 1,186(20.3%) |
| Yes | 1,901(56.5%) | 4,662(79.7%) |
| **Had books at home(at age 10)** |  |  |
| No | 2,394(71.2%) | 3,133(53.6%) |
| Yes | 969(28.8%) | 2,715(46.4%) |
| **Economic situation (until age 15)** |  |  |
| Above average | 109(3.2%) | 262(4.5%) |
| Average | 637(18.9%) | 1,462(25.0%) |
| Poor | 2,315(68.8%) | 3,370(57.6%) |
| Highly variable | 302(9.0%) | 754(12.9%) |
| **Health condition(until age 15)** |  |  |
| Excellent/very good | 690(20.5%) | 1,435(24.5%) |
| Good | 2,095(62.3%) | 3,340(57.1%) |
| Fair | 393(11.7%) | 780(13.3%) |
| Bad | 185(5.5%) | 293(5.0%) |
| **Absent from school due to health issues(until age 15)** |  |  |
| No | 2,118(63.0%) | 4,525(77.4%) |
| Yes | 242(7.2%) | 504(8.6%) |
| Did not go to school until 15 | 1,003(29.8%) | 819(14.0%) |
| **Common childhood infectious diseases(until age 15)** |  |  |
| No | 689(20.5%) | 851(14.6%) |
| Yes | 2,674(79.5%) | 4,997(85.4%) |
| **Poliomyelitis(until age 15)** |  |  |
| No | 3,322(98.8%) | 5,781(98.9%) |
| Yes | 41(1.2%) | 67(1.1%) |
| **Respiratory diseases(until age 15)** |  |  |
| No | 3,139(93.3%) | 5,365(91.7%) |
| Yes | 224(6.7%) | 483(8.3%) |
| **Allergy(until age 15)** |  |  |
| No | 3,137(93.3%) | 5,220(89.3%) |
| Yes | 226(6.7%) | 628(10.7%) |
| **Severe diarrhea (typhoid or other until age 15)** |  |  |
| No | 3,040(90.4%) | 5,086(87.0%) |
| Yes | 323(9.6%) | 762(13.0%) |
| **Chronic ear problems(until age 15)** |  |  |
| No | 3,218(95.7%) | 5,528(94.5%) |
| Yes | 145(4.3%) | 320(5.5%) |
| Note: Median (Q_1_,Q_3_); Common childhood infectious diseases include measles, rubella, chickenpox, mumps, diphtheria, whooping cough, and scarlet fever. | | |

**Supplementary Table 2**

Characteristics of study participants stratified by recent vaccination.

| **Characteristic** | **recent vaccination** | |
| --- | --- | --- |
|  | **Unvaccinated**  **(n = 2,823)** | **Vaccinated**  **(n = 7,040)** |
| **Age** | 59 (55-68) | 67 (60-75) |
| **Sex** |  |  |
| Male | 1,557(55.2%) | 4,288(60.9%) |
| Female | 1,266(44.8%) | 2,752(39.1%) |
| **Rural residence until age 15** |  |  |
| No | 1,236(43.8%) | 2,993(42.5%) |
| Yes | 1,587(56.2%) | 4,047(57.5%) |
| **Marriage** |  |  |
| Single | 428(15.2%) | 769(10.9%) |
| Married/ Common-law marriage/live together | 1,565(55.4%) | 3,686(52.4%) |
| Divorced or separated | 355(12.6%) | 881(12.5%) |
| Widow(er) | 475(16.8%) | 1,704(24.2%) |
| **Number of children** | 3 (2,4) | 3 (2,5) |
| **Mother's educational level** |  |  |
| Illiterate/never studied | 1,665(59.0%) | 4,230(60.1%) |
| Incomplete elementary school | 660(23.4%) | 1,582(22.5%) |
| Completed elementary school | 349(12.4%) | 795(11.3%) |
| Completed middle school | 69(2.4%) | 233(3.3%) |
| Completed High school or above | 80(2.8%) | 200(2.8%) |
| **Father's educational level** |  |  |
| Illiterate/never studied | 1,528(54.1%) | 3,796(53.9%) |
| Incomplete elementary school | 699(24.8%) | 1,692(24.0%) |
| Completed elementary school | 351(12.4%) | 912(13.0%) |
| Completed middle school | 83(2.9%) | 265(3.8%) |
| Completed High school or above | 162(5.7%) | 375(5.3%) |
| **Educational background** |  |  |
| Never studied | 436(15.4%) | 1,131(16.1%) |
| Incomplete elementary school | 1,267(44.9%) | 3,392(48.2%) |
| Completed elementary school | 388(13.7%) | 839(11.9%) |
| Completed middle school | 147(5.2%) | 244(3.5%) |
| Completed high school | 441(15.6%) | 1,037(14.7%) |
| College degree or above | 144(5.1%) | 397(5.6%) |
| **Discrimination (when seeking medical services)** |  |  |
| No | 2,675(94.8%) | 6,706(95.3%) |
| Yes | 148(5.2%) | 334(4.7%) |
| **Economic situation (until age 15)** |  |  |
| Was above average | 113(4.0%) | 290(4.1%) |
| Was about average | 622(22.0%) | 1,583(22.5%) |
| Was poor | 1,760(62.3%) | 4,375(62.1%) |
| Was varied a lot | 328(11.6%) | 792(11.3%) |
| **Common childhood infectious diseases(until age 15)** |  |  |
| No | 505(17.9%) | 1,137(16.2%) |
| Yes | 2,318(82.1%) | 5,903(83.8%) |
| **Poliomyelitis(until age 15)** |  |  |
| No | 2,778(98.4%) | 6,970(99.0%) |
| Yes | 45(1.6%) | 70(1.0%) |
| **Respiratory diseases(until age 15)** |  |  |
| No | 2,633(93.3%) | 6,481(92.1%) |
| Yes | 190(6.7%) | 559(7.9%) |
| **Allergy (until age 15)** |  |  |
| No | 2,578(91.3%) | 6,388(90.7%) |
| Yes | 245(8.7%) | 652(9.3%) |
| **Typhoid(until age 15)** |  |  |
| No | 2,492(88.3%) | 6,228(88.5%) |
| Yes | 331(11.7%) | 812(11.5%) |
| **Vaccination(until age 15)** |  |  |
| No | 894(31.7%) | 2,750(39.1%) |
| Yes | 1,929(68.3%) | 4,290(60.9%) |
| **Economic situation (relative to before adulthood)** |  |  |
| Improved | 2,269(80.4%) | 5,764(81.9%) |
| Remained the same | 365(12.9%) | 875(12.4%) |
| Got worse | 189(6.7%) | 401(5.7%) |
| **Have a monetary job(within 30 days)** |  |  |
| No | 1,762(62.4%) | 5,555(78.9%) |
| Yes | 1,061(37.6%) | 1,485(21.1%) |
| **Life satisfaction(income/work)** | 5 (4-7) | 5 (4-7) |
| **Exercise frequency** |  |  |
| None | 1,815(64.3%) | 4,661(66.2%) |
| 1 day | 132(4.7%) | 390(5.5%) |
| 2 days | 226(8.0%) | 471(6.7%) |
| 3 days | 194(6.9%) | 499(7.1%) |
| 4 days | 108(3.8%) | 207(2.9%) |
| 5 days | 136(4.8%) | 281(4.0%) |
| 6 days | 40(1.4%) | 115(1.6%) |
| 7 days | 172(6.1%) | 416(5.9%) |
| **Alcohol intake frequency** |  |  |
| Never | 2,060(73.0%) | 5,658(80.4%) |
| Less than once a month | 280(9.9%) | 591(8.4%) |
| Once a month or more | 483(17.1%) | 791(11.2%) |
| **Health condition** |  |  |
| Very good or excellent | 198(7.0%) | 415(5.9%) |
| Good | 1,128(40.0%) | 2,757(39.2%) |
| Regular | 1,030(36.5%) | 2,669(37.9%) |
| Bad | 349(12.4%) | 892(12.7%) |
| Very bad | 118(4.2%) | 307(4.4%) |
| **Frequency of poor mental health** | 0 (0-1) | 0 (0-0) |
| **Hypertension** |  |  |
| No | 1,628(57.7%) | 3,124(44.4%) |
| Yes | 1,195(42.3%) | 3,916(55.6%) |
| **Diabetes** |  |  |
| No | 2,478(87.8%) | 5,605(79.6%) |
| Yes | 345(12.2%) | 1,435(20.4%) |
| **Depression** |  |  |
| No | 2,471(87.5%) | 6,125(87.0%) |
| Yes | 352(12.5%) | 915(13.0%) |
| **Cancer** |  |  |
| No | 2,725(96.5%) | 6,720(95.5%) |
| Yes | 98(3.5%) | 320(4.5%) |
| **Sleep quality** |  |  |
| Very good | 396(14.0%) | 842(12.0%) |
| Good | 1,291(45.7%) | 3,439(48.8%) |
| Regular | 648(23.0%) | 1,702(24.2%) |
| Bad | 381(13.5%) | 837(11.9%) |
| Very bad | 107(3.8%) | 220(3.1%) |
| **Sleep problems (last 6 months)** |  |  |
| No | 2,194(77.7%) | 5,664(80.5%) |
| Yes | 629(22.3%) | 1,376(19.5%) |
| **Have friends** |  |  |
| No | 305(10.8%) | 704(10.0%) |
| Yes | 2,518(89.2%) | 6,336(90.0%) |
| **Optimistic about the future** |  |  |
| Never | 190(6.7%) | 399(5.7%) |
| Sometimes | 1,086(38.5%) | 2,617(37.2%) |
| Always | 1,547(54.8%) | 4,024(57.2%) |
| **BMI** | 27.1 (24.0,30.8) | 27.3 (24.2,30.8) |
| Note: Median (Q_1_,Q_3_); Common childhood infectious diseases include measles, rubella, chickenpox, mumps, diphtheria, whooping cough, and scarlet fever. | | |

**Supplementary Table 3**

Predictors of childhood vaccination identified by both multivariable logistic regression and machine learning variable importance rankings.

| **Variable Type** | **Predictor** | **uOR (95% CI),**  **p value** | **aOR (95% CI),**  **p value** | **ML rank**  **（GLM/ GBM/ DRF/MLP）** | **Best ML rank** | **Top10 models** | **Category** |
| --- | --- | --- | --- | --- | --- | --- | --- |
| Sociodemographic | Age | 0.95 (0.94-0.95)，p<0.001 | 0.96 (0.95-0.96)，p<0.001 | 1/1/1/1 | 1 (GLM) | 4/4 | Concordant |
|  | Sex | 1.14 (1.05-1.24)，p=0.003 | 1.14 (1.04-1.26)，p=0.005 | 17/8/8/8 | 8 (GBM) | 3/4 | Concordant |
|  | Rural residence until 15 | 0.51 (0.46-0.55)，p<0.001 | 0.75 (0.67-0.83)，p<0.001 | 7/10/11/7 | 7 (GLM) | 3/4 | Concordant |
|  | Mother's educational level: Completed elementary school | 3.34 (2.85-3.92)，p<0.001 | 1.81 (1.48-2.20)，p<0.001 | 6/6/6/4 | 4 (MLP) | 4/4 | Concordant |
|  | Father's educational level: Incomplete elementary school | 1.86 (1.67-2.06)，p<0.001 | 1.22 (1.07-1.39)，p=0.003 | 8/5/5/14 | 5 (GBM) | 3/4 | Concordant |
|  | Age when starting school | 1.01 (1.00-1.02)，p=0.023 | 1.00 (0.99-1.00)，p=0.429 | 15/2/2/3 | 2 (GBM) | 3/4 | Prediction-only |
|  | Schooling at age 10 | 3.02 (2.75-3.32)，p<0.001 | 1.69 (1.49-1.91)，p<0.001 | 2/9/7/6 | 2 (GLM) | 4/4 | Concordant |
| Environmental | Lived with family (at age 10) | 1.21 (0.99-1.48)，p=0.064 | 1.07 (0.86-1.33)，p=0.552 | 16/16/16/16 | 16 (GLM) | 0/4 | Neither |
|  | Had books at home(at age 10) | 2.14 (1.96-2.34)，p<0.001 | 1.48 (1.33-1.64)，p<0.001 | 4/12/10/13 | 4 (GLM) | 2/4 | Concordant |
|  | Economic situation (until age 15): Poor | 0.61 (0.48-0.76)，p<0.001 | 0.81 (0.63-1.03)，p=0.09 | 18/4/4/9 | 4 (GBM) | 3/4 | Prediction-only |
| Lifestyle | Health condition(until age 15): Good | 0.77 (0.69-0.85)，p<0.001 | 0.93 (0.82-1.04)，p=0.189 | 14/3/3/10 | 3 (GBM) | 3/4 | Prediction-only |
|  | Absent from school due to health issues(until age 15): Did not go to school until 15 | 0.38 (0.34-0.42)，p<0.001 | 0.85 (0.74-0.98)，p=0.021 | 10/7/9/5 | 5 (MLP) | 4/4 | Concordant |
| Disease | Common childhood infectious diseases(until age 15) | 1.26 (1.07-1.49)，p=0.006 | 1.06 (0.88-1.27)，p=0.541 | 3/11/12/18 | 3 (GLM) | 1/4 | Prediction-only |
|  | Poliomyelitis(until age 15) | 1.67 (1.43-1.96)，p<0.001 | 1.37 (1.15-1.64)，p<0.001 | 11/18/18/2 | 2 (MLP) | 1/4 | Concordant |
|  | Respiratory diseases(until age 15) | 1.28 (1.05-1.57)，p=0.015 | 1.15 (0.92-1.43)，p=0.219 | 12/15/15/15 | 12 (GLM) | 0/4 | Neither |
|  | Allergy (until age 15) | 1.51 (1.35-1.69)，p<0.001 | 1.64 (1.46-1.86)，p<0.001 | 9/14/14/17 | 9 (GLM) | 1/4 | Concordant |
|  | Severe diarrhea (typhoid or other until age 15) | 0.94 (0.64-1.39)，p=0.753 | -- | 5/13/13/12 | 5 (GLM) | 1/4 | Prediction-only |
|  | Chronic ear problems (until age 15) | 1.41 (1.23-1.62)，p<0.001 | 1.55 (1.33-1.81)，p<0.001 | 13/17/17/11 | 11 (MLP) | 0/4 | Inference-only |

Note: Predictors are categorized based on agreement between logistic regression (LR) and machine learning (ML) models:uOR:unadjusted odds ratio;aOR:adjusted odds ratios;CI:Confidence Interval; GLM:Generalized Linear Model; GBM:Gradient Boosting Machine;DRF:Decision Tree Regression;MLP:Multilayer Perceptron;Concordant: Statistically significant in LR (p < 0.05) AND ranked in top 10 by at least one ML model.Inference-only: Statistically significant in LR but NOT ranked in top 10 by any ML model.Prediction-only: Not significant in LR (or excluded) but ranked in top 10 by at least one ML model.Neither: Not significant in LR and not ranked in top 10 by any ML model.

**Supplementary Table 4**

Predictors of recent vaccination identified by both multivariable logistic regression and machine learning variable importance rankings.

| **Variable Type** | **Predictor** | **uOR (95% CI),**  **p value** | **aOR (95% CI),**  **p value** | **ML rank**  **(GLM/GBM/DRF)** | **Best ML rank** | **Top10 models** | **Category** |
| --- | --- | --- | --- | --- | --- | --- | --- |
| Sociodemographic | Age | 1.06 (1.06-1.07)，p<0.001 | 1.06 (1.05-1.06)，p<0.001 | 1/1/1 | 1 (GLM) | 3/3 | Concordant |
|  | Sex | 0.79 (0.72-0.86)，p<0.001 | 0.84 (0.76-0.93)，p<0.001 | 28/19/20 | 19 (GBM) | 0/3 | Inference-only |
|  | Rural residence until age 15 | 1.05 (0.96-1.15)，p=0.25 | -- | 29/18/19 | 18 (GBM) | 0/3 | Neither |
|  | Marriage: Married/ Common-law marriage/live together | 1.31 (1.15-1.50)，p<0.001 | 1.29 (1.12-1.50)，p<0.001 | 13/10/9 | 9 (DRF) | 2/3 | Concordant |
|  | Number of children | 1.04 (1.02-1.06)，p<0.001 | 0.97 (0.95-0.99)，p=0.009 | 17/4/4 | 4 (GBM) | 2/3 | Concordant |
|  | Mother's educational level: Completed middle school | 1.33 (1.01-1.75)，p=0.042 | 1.57 (1.17-2.11)，p=0.003 | 5/13/10 | 5 (GLM) | 2/3 | Concordant |
|  | Father's educational level: Completed elementary school | 1.29 (1.00-1.66)，p=0.052 | -- | 31/11/13 | 11 (GBM) | 0/3 | Neither |
|  | Educational background: Completed high school | 1.03 (0.91-1.17)，p=0.629 | 1.31 (1.14-1.51)，p<0.001 | 16/7/8 | 7 (GBM) | 2/3 | Concordant |
|  | Economic situation (relative to before adulthood): Got worse | 0.84 (0.70-1.00)，p=0.049 | -- | 32/17/18 | 17 (GBM) | 0/3 | Neither |
|  | Have a monetary job(within 30 days) | 0.44 (0.40-0.49)，p<0.001 | 0.67 (0.60-0.75)，p<0.001 | 2/15/15 | 2 (GLM) | 1/3 | Concordant |
|  | Life satisfaction(income/work) | 1.02 (1.00-1.04)，p=0.054 | 1.02 (1.00-1.04)，p=0.067 | 27/3/3 | 3 (GBM) | 2/3 | Prediction-only |
|  | Health condition: Very bad | 1.24 (1.03-1.49)，p=0.024 | -- | 12/8/7 | 7 (DRF) | 2/3 | Prediction-only |
|  | Have friends | 1.09 (0.95-1.26)，p=0.234 | -- | 20/28/27 | 20 (GLM) | 0/3 | Neither |
| Environmental | Discrimination (when seeking medical services) | 0.90 (0.74-1.10)，p=0.3 | -- | 26/30/30 | 26 (GLM) | 0/3 | Neither |
|  | Economic situation (until age 15): Was varied a lot | 0.94 (0.73-1.21)，p=0.636 | -- | 23/12/11 | 11 (DRF) | 0/3 | Neither |
| Lifestyle | Exercise frequency | 0.88 (0.78-0.99)，p=0.035 | 1.04 (0.91-1.18)，p=0.583 | 11/6/6 | 6 (GBM) | 2/3 | Prediction-only |
|  | Alcohol intake frequency: Once a month or more | 0.60 (0.53-0.67)，p<0.001 | 0.79 (0.69-0.91)，p<0.001 | 6/16/16 | 6 (GLM) | 1/3 | Concordant |
|  | Frequency of poor mental health | 0.99 (0.99-1.00)，p=0.02 | 0.99 (0.99-1.00)，p=0.038 | 4/9/12 | 4 (GLM) | 2/3 | Concordant |
|  | Sleep quality: Regular | 1.25 (1.09-1.43)，p=0.001 | 1.23 (1.06-1.42)，p=0.005 | 15/5/5 | 5 (GBM) | 2/3 | Concordant |
|  | Sleep problems (last 6 months) | 0.85 (0.76-0.94)，p=0.002 | 0.87 (0.76-0.99)，p=0.038 | 30/24/22 | 22 (DRF) | 0/3 | Inference-only |
|  | Optimistic about the future: Always | 1.24 (1.03-1.49)，p=0.021 | 1.37 (1.12-1.67)，p=0.002 | 10/14/14 | 10 (GLM) | 1/3 | Concordant |
| Disease | Respiratory diseases(until age 15) | 1.20 (1.01-1.42)，p=0.041 | 1.22 (1.02-1.46)，p=0.029 | 22/29/29 | 22 (GLM) | 0/3 | Inference-only |
|  | Allergy (until age 15) | 1.07 (0.92-1.25)，p=0.363 | -- | 18/25/28 | 18 (GLM) | 0/3 | Neither |
|  | Common childhood infectious diseases(until age 15) | 1.13 (1.01-1.27)，p=0.036 | 1.03 (0.91-1.16)，p=0.666 | 21/22/23 | 21 (GLM) | 0/3 | Neither |
|  | Poliomyelitis(until age 15) | 0.62 (0.43-0.90)，p=0.013 | 0.72 (0.48-1.07)，p=0.102 | 14/32/32 | 14 (GLM) | 0/3 | Neither |
|  | Severe diarrhea(until age 15) | 0.98 (0.86-1.12)，p=0.789 | -- | 24/26/26 | 24 (GLM) | 0/3 | Neither |
|  | Vaccination(until age 15) | 0.72 (0.66-0.79)，p<0.001 | 0.91 (0.82-1.00)，p=0.06 | 9/21/21 | 9 (GLM) | 1/3 | Prediction-only |
|  | Hypertension | 1.71 (1.56-1.87)，p<0.001 | 1.35 (1.23-1.49)，p<0.001 | 3/20/17 | 3 (GLM) | 1/3 | Concordant |
|  | Diabetes | 1.84 (1.62-2.09)，p<0.001 | 1.56 (1.37-1.79)，p<0.001 | 8/23/24 | 8 (GLM) | 1/3 | Concordant |
|  | Depression | 1.05 (0.92-1.20)，p=0.479 | -- | 7/27/25 | 7 (GLM) | 1/3 | Prediction-only |
|  | Cancer | 1.32 (1.05-1.67)，p=0.017 | 1.09 (0.86-1.39)，p=0.471 | 19/31/31 | 19 (GLM) | 0/3 | Neither |
| Physical exams | BMI | 1.00 (0.99-1.01)，p=0.475 | -- | 25/2/2 | 2 (GBM) | 2/3 | Prediction-only |

Note: Predictors are categorized based on agreement between logistic regression (LR) and machine learning (ML) models:uOR:unadjusted odds ratios;aOR:adjusted odds ratios;CI:Confidence Interval; GLM:Generalized Linear Model; GBM:Gradient Boosting Machine;DRF:Decision Tree Regression;Concordant: Statistically significant in LR (p < 0.05) AND ranked in top 10 by at least one ML model.Inference-only: Statistically significant in LR but NOT ranked in top 10 by any ML model.Prediction-only: Not significant in LR (or excluded) but ranked in top 10 by at least one ML model.Neither: Not significant in LR and not ranked in top 10 by any ML model.

**Supplementary Table 5.**

Class distribution before and after training-set class balancing

| Outcome | Dataset | No | Yes | Total |
| --- | --- | --- | --- | --- |
| Childhood vaccination | Overall analytic sample | 3,363 (36.5%) | 5,848 (63.5%) | 9,211 |
| Childhood vaccination | Original training set | 2,691 (36.5%) | 4,679 (63.5%) | 7,370 |
| Childhood vaccination | Balanced training set | 2,691 (50.0%) | 2,691 (50.0%) | 5,382 |
| Childhood vaccination | Testing set | 672 (36.5%) | 1,169 (63.5%) | 1,841 |
| Recent influenza vaccination | Overall analytic sample | 2,823 (28.6%) | 7,040 (71.4%) | 9,863 |
| Recent influenza vaccination | Original training set | 2,259 (28.6%) | 5,632 (71.4%) | 7,891 |
| Recent influenza vaccination | Balanced training set | 2,259 (50.0%) | 2,259 (50.0%) | 4,518 |
| Recent influenza vaccination | Testing set | 564 (28.6%) | 1,408 (71.4%) | 1,972 |

**Supplementary Fig. 1**. Multivariable logistic regression forest plot for childhood vaccination

**Supplementary Fig. 2.** Multivariable logistic regression forest plot for recent vaccination
